# Ascorbic Acid-Modified Silicones: Crosslinking and Antioxidant Delivery

**DOI:** 10.3390/polym14225040

**Published:** 2022-11-21

**Authors:** Guanhua Lu, Akop Yepremyen, Khaled Tamim, Yang Chen, Michael A. Brook

**Affiliations:** Department of Chemistry and Chemical Biology, McMaster University, 1280 Main St. W., Hamilton, ON L8S 4M1, Canada

**Keywords:** ascorbic acid, silicone elastomer, antioxidant activity, reductive cleavage, aza-Michael addition

## Abstract

Vitamin C is widely used as an antioxidant in biological systems. The very high density of functional groups makes it challenging to selectively tether this molecule to other moieties. We report that, following protection of the enediol as benzyl ethers, the introduction of an acrylate ester at C1 is straightforward. Ascorbic acid-modified silicones were synthesized via aza-Michael reactions of aminoalkylsilicones with ascorbic acrylate. Viscous oils formed when the amine/acrylate ratios were <1. However, at higher amine/acrylate ratios with pendent silicones, a double reaction occurred to give robust elastomers whose modulus is readily tuned simply by controlling the ascorbic acid amine ratio that leads to crosslinks. Reduction with H_2_/Pd removed the benzyl ethers and led to increased crosslinking, and either liberated the antioxidant small molecule or produced antioxidant elastomers. These pro-antioxidant elastomers show the power of exploiting natural materials as co-constituents of silicone polymers.

## 1. Introduction

Antioxidants are needed by both biological and synthetic materials for protection against the detrimental effects of oxidative radical species [1,2,3,4,5,6,7]. Their presence has been demonstrated to preserve the mechanical and other properties of polymers, especially in high oxidative stress environments, including high temperatures or biological environments. Frequently, antioxidants are simply added to a material and their efficacy and longevity depend both on their specific chemistry—their response to oxidative stress [4,7]—and whether they leach from the material to adjacent media [7,8]. Covalently attaching antioxidants to polymer matrices avoids the latter problem [9,10,11,12,13,14,15]. Some simple examples of grafted antioxidants include gallic acid or catechin grafted to gelatin [11], and the use of grafted phenolic antioxidants on fuel cells [12] or polyisobutylene [10].

Silicone polymers well known for their biocompatibility, electrical resistance, thermostability, and high oxidative resistance [16]; they are redox insensitive. However, in many of their applications, there would be a benefit if they could convey antioxidant activity to adjacent materials. For example, biomaterial applications ranging from topical contact lenses/cosmetics products to implanted biomaterials such as breast implants and catheters would benefit from the presence of antioxidants [17]. However, release of any bioactive from the silicone polymer could be disadvantageous [18]. 

Leivo et al. demonstrated the use of ascorbic acid as a linker between amine-modified silicone elastomer surfaces and collagen for cell culture [19]. The enediol was involved in forming one imine with amines from each entity and, eventually, undergoing oxidative cleavage and ceasing to function as an antioxidant. In essence, ascorbic acid was analogous to, but less toxic than, glutaraldehyde because both can react twice with an amine to form imines. 

Our objective was to graft ascorbic acid to silicones while maintaining antioxidant activity. Ascorbic acid/vitamin C was chosen as the candidate antioxidant modification to graft to silicones because of its robust antioxidant and antiviral properties [1,5,20,21], which may be due to its redox properties [22]. It is found in a wide variety of fresh vegetables and fruits, and at the highest concentrations in citrus fruits and green leafy vegetables [3]. Due to the hydrophilic nature of ascorbic acid, it is challenging to incorporate it into very low-energy, hydrophobic silicone matrices. We report the formation of more hydrophobic, protected ascorbic acid-modified silicones that can be crosslinked and, when desired, deprotected with concomitant release or generation of antioxidants. 

## 2. Materials and Methods

### 2.1. Materials

Potassium carbonate, sodium sulfate benzyl bromide, acryloyl chloride, triethylamine, ascorbic acid (vitamin C), deuterated methanol (MeOH-d_4_), deuterated chloroform (CDCl_3_), Pd/C (palladium, 5%wt. % (dry basis) on activated carbon), EtOAc, hexanes, DMF and all other solvents were purchased from Sigma Aldrich (Burlington, MA, USA). H_2_ (ultra-high purity 5.0) was taken from a Praxair gas cylinder. Telechelic aminopropylsilicone **T334** (DMS-A31, 0.11–0.12% mol aminopropylmethylsiloxane, molar mass ~25,000 g mol^−1^); a lower molar mass **P21** (AMS-152, 4–5% mol aminopropylmethylsiloxane, molar mass ~8000 g mol^−1^) pendent silicone and an analogous higher mass material **P22** (AMS-1203, 20–25% mol aminopropylmethylsiloxane, molar mass ~20,000 g mol^−1^) were purchased from Gelest (Morrisville, PA, USA). 

### 2.2. Methods

^1^H NMR spectra were recorded on Bruker NEO 600 MHz or NEO 500 MHz nuclear magnetic resonance spectrometer. A Shore OO durometer (Rex Gauge Company, Inc., Buffalo Grove, IL, USA) was used to characterize the hardness of the elastomer. A centrifuge was used for sedimentation of charcoal during purification of the hydrogenated product.

### 2.3. Synthesis of Benzyl-Protected Ascorbic Acid and Modification with Acrylate 1

Ascorbic acid (6.0 g, 34 mmol) was dissolved in DMF (20 mL). K_2_CO_3_ (11.8 g, 85 mmol) was added and the mixture was stirred for 1 h at 50 °C. A solution of benzyl bromide (12 g, 70 mmol) in DMF (15 mL) was added dropwise to the ascorbic acid mixture and stirred for 5 h at room temperature under an N_2_ blanket. The reaction solution was filtered through a pad of Celite and washed with ethyl acetate. The combined organic phases were extracted with H_2_O (3 × 100 mL). The organic layer was collected, dried over Na_2_SO_4_, and filtered. Following concentration by rotary evaporation, the crude product was purified by flash column chromatography (hexanes: EtOAc 1:3 to 1:1) to afford benzylated ascorbic acid (4.3 g, 36%) as light-yellow oil (for NMR and mass spectrum, see Supporting Information SI). ^1^H NMR (CDCl_3_, 600 MHz): 7.41–7.16 (m, 10H), 5.46–5.05 m, 4H), 4.68 (d, *J* = 2.0 Hz, 1H), 3.91 (m, 1H), 3.82–3.70 (m, 2H). 

A stirred solution of the benzylated product (4.01 g, 11.3 mmol) was added to anhydrous CH_2_Cl_2_ (50 mL) and stirred over ice for 10 min. Et_3_N (1.57 mL, 11.3 mmol) was added to the reaction mixture and let stir for 5 min. Acryloyl chloride (0.91 mL, 11.3 mmol) was first dissolved in 10 mL of anhydrous CH_2_Cl_2_ and added into the reaction mixture dropwise over 1 h. The reaction was stirred for 5 h at 0 °C and filtered over Celite. The organic layer was washed with brine (3 × 40 mL), dried over (Na_2_SO_4_), and filtered. Following concentration, the crude product was purified by flash column chromatography (hexanes: EtOAc 2:1) to afford **1** (858 mg, 19%) as a white solid (for NMR, see Supporting Information). Note: the isolated yield was low due to the formation of di-adduct at C2 of ascorbic acid (in addition to C1) that was difficult to separate from the monoadduct; the isolated yield of the mixture of mono- and diadducts was 77%. 

^1^H NMR (CDCl_3,_ 600 MHz): 7.52–7.29 (m, 10H), 6.57 (dd, *J* = 17.2, 1.5 Hz, 1H), 6.25 (dd, *J* = 17.2, 10.3 Hz, 1H), 5.99 (dd, *J* = 10.3, 1.5 Hz, 1H), 5.38-5.18 (m, 4H), 4.83 (d, *J =* 1.9 Hz, 1H), 4.49 (qd, *J* = 11.5, 6.2 Hz, 2H), 4.33–4.17 (m, 1H), 3.06 (br, 1H).

### 2.4. Reactions with Benzylated Acryl Ascorbic Acid 1 by Butylamine (Bn2AA)

Benzylated acryl ascorbic acid **1** (0.026 g, 0.06 mmol) and excess butylamine (0.3 g, 4 mmol) were mixed neat and stirred overnight. The product mixture was concentrated over vacuum and dried under nitrogen for 2 h before NMR was taken (SI). 

### 2.5. Benzylated, Acryl Ascorbic Acid-Modified Silicones 

#### 2.5.1. Telechelic Silicone

Benzylated acryl ascorbic acid **1** (0.05 g, 0.12 mmol) and **T334** (1.16, 0.12 mmol amine) were dissolved in IPA (5 mL) and stirred overnight. The reaction solution was concentrated, and a yellow oil was obtained (SI). 

#### 2.5.2. Pendant Silicones

Benzylated acryl ascorbic acid **1** (0.4g, 0.96 mmol) was first dissolved in IPA (20 mL) to generate a 0.02 mg/mL stock solution. The stock solution (2.5 mL) and different quantities of **P22** (2% **P22-2**, 5% **P22-5**, 10% **P22-10**, 15% **P22-15**, 20% **P22-20**, 50% **P22-50**, 75% **P22-75**, and 100% **P22-100**) and **P21** (25% **P21-25**) (Table S1), in quantities based on 1:1 amine:acrylate) were added and stirred in additional IPA (5 mL total volume) overnight; the product solution was concentrated by evaporating the solvent in oven overnight at 50 °C. NMR was taken for resulting yellow oil (SI). 

### 2.6. Debenzylation (Hydrogenation) of Benzylated Ascorbic Acid Silicones 

Hydrogenation was performed by first dissolving benzylated ascorbic acid silicone **P22-20** (0.049 g, 0.12 mmol) in IPA (50 mL) in a 100 mL round-bottomed flask equipped with stir bar. Based on the benzyl group 15% mole Pd (0.039 g 10% Pd/C, 0.037 mmol) was then added to the solution. The round-bottomed flask was then connected to a dual manifold, after 10 × de-gas/ nitrogen purges, the manifold was then connected to an H_2_ balloon; the system was then 5 × de-gas/hydrogen purged before switching to hydrogen overnight. After the reaction was performed, the solution was then centrifuged at 14,000 rpm for 30 min to give a slightly grey solution that was vacuum filtered through a Celite plug followed by concentration using rotary evaporation, the resulting clear oil was then washed with CDCl_3_ and centrifuged at 14,000 rpm for 5 min. Two phases resulted: a CDCl_3_ phase (from which NMR was measured) supernatant and a cloudy oil. MeOH-d_4_ was added to the oily residue with shaking. After centrifugation at 14,000 rpm for 5 min, the solution phase was collected and the NMR spectrum was recorded, the remaining solid (ascorbic acid) was then dissolved in D_2_O and an NMR spectrum was recorded (SI). 

For most compounds, however, including **P22-10**, and **P21-25**, the reduction was accompanied by a change in color: **P22-2** went from a pale-yellow oil to a brown oil; **P22-10** and **P21-25** yielded black elastomers. 

### 2.7. Kinetic Study of Benzyl Acryl Ascorbic Acid and Pendant Silicone

A kinetic study was conducted using NMR. Benzylated acryl ascorbic acid (0.05 g, 0.12 mmol) was first dissolved in deuterated chloroform or MeOH-d_4_ (0.35 mL); **P22** (0.049 g, 0.12 mmol) was dissolved separately in deuterated chloroform or MeOH-d_4_ (0.35 mL). The two components were combined right before the first NMR spectrum was collected at time 0 min, and then at 0.5 h, 1 h, 2 h, 4 h, 8 h, 12 h, and 24 h to monitor the reaction process (SI). 

### 2.8. DPPH Assay for Elastomer Samples

For quantitative analyses, the debenzylated products of **P21-25** (84.6 mM, based on the concentration of ascorbic acid in 50 mg of **P21**) were swelled in IPA (1 mL) in a 1.5 mL centrifuge tube in quantities (Table S2); the sample was allowed to swell for 2 h. The DPPH solution (0.5 mL of 0.2 mM) was then added to the sample and the mixture sat in the dark for 30 min to react. The resulting solution was then filtered, and 200 μL aliquot of the resulting solution was added to a 96-well plate in triplicate. Scans were taken for each well at 520 nm from the plate reader and the results were recorded (Table S3). **P21-25** and **P22-2** samples, after hydrogenation, were similarly treated. **P22-10** and **P21-25** elastomers showed moderate antioxidant activity, whereas **P22-2** showed no significant antioxidant activity. 

### 2.9. DPPH Assay of Ascorbic Acid and Benzylated Ascorbic Acid Control

DPPH assays were performed for ascorbic acid (AA) and benzylated ascorbic acid as controls to the ascorbic acid-modified elastomer samples. Stock solutions were prepared by dissolving ascorbic acid (74.5 mg) in DI water (5 mL) or benzylated ascorbic acid (150.7 mg) in IPA (5 mL), respectively. The stock solution was then diluted 2-fold, 4-fold, 10-fold, or 20-fold. Each concentration (0.5 mL) of the control solution was added to a 1.5 mL centrifuge tube, subsequently (0.5 mL of 0.2 mM DPPH solution) was added to the tube, mixed, and allowed to rest in the dark for 30 min. An aliquot (200 μL) of the resulting solution was added to a 96-well plate in triplicate. Scans were taken for each well at 520 nm from the plate reader and the results were recorded (Table S3). 

## 3. Results

### 3.1. Synthesis of Benzyl-Protected Ascorbic Acid and Modification with Acrylate

**Figure 1:**
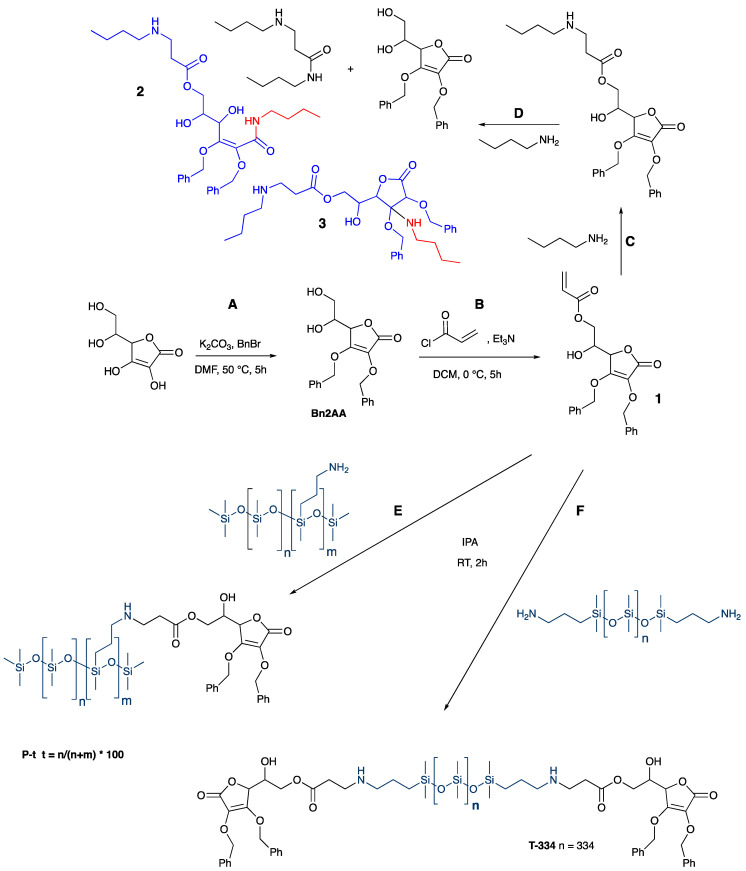
Synthesis of (**A**,**B**): benzyl protected, acrylated ascorbic acid and conversion to (**C**): mono or (**D**): dibutyl amine derivatives, or conversion to (**E**): pendent, or (**F**): telechelic ascorbic acid-modified silicone polymers.

Survey experiments demonstrated that ascorbic acid (AA) was both too polar and too reactive, in particular to oxidation, for many of the desired synthetic processes to succeed. Therefore, the enols in AA were protected as benzyl ethers using a simple Williamson approach (Figure 1A). Acrylic ester formation using acryloyl chloride preferentially occurred at the primary alcohol to give **1** (Figure 1B); no secondary alcohol modification was observed, as shown by NMR (SI).

### 3.2. Benzylated, Acryl Ascorbic Acid-Modified Silicones and Cleavage of Acylated, Benzylated Ascorbic Acid by Butyl Amine

Under oxidizing conditions, ascorbic acid can be induced to react twice with amines to (putatively) form a 1,2-dimine from dienol [19]. Model studies were undertaken with the protected derivative **1** to understand how the functional differences with the protected compound would manifest when aminosilicones were present. ^1^H NMR showed that two equivalents of butylamine also reacted with benzylated acryl ascorbic acid **1**: the first performed an aza-Michael addition with the acrylate; and the second led to amidation and cleavage of the aza-Michael acrylate (Figure 1C,D and Supporting Information). Other motifs are also likely involved, including ring-opening cleavage or secondary Michael additions (**2**, **3**). It was, therefore, anticipated that linear silicone oils, modified with ascorbic acid, would arise from aza-Michael reactions between **1** and aminoalkylsilicones provided that the stoichiometry of [H_2_N]/[acrylate] was kept below 1:1. 

The aza-Michael process was both trivial and facile, requiring only stirring in IPA (isopropanol). A library of ascorbic acid-modified silicones could then be prepared from this key functional molecule **1** by the aza-Michael reaction with either pendent (Figure 1E) or telechelic (Figure 1F) aminoalkylsilicones containing different amine densities. 

The telechelic sample **T334** (nomenclature: **Tn**, where n is the number of Me_2_SiO units in the chain, **T334**, n = 334 Figure 1) was modified completely at both termini with **1**. With the pendent silicones both partial **P22-x** (nomenclature: **Pt-x** where t is the % of aminopropyl monomers m in the chain (m/(m + n) × 100, normally t = 22, and x = 2, 5, 10, 15, 20, 50, 75, and 100, Figure 1) and complete modification **P22-100** with **1** was performed. The telechelic products and pendent products made with lower equivalents of AA (**P22-2** ➞ **P22-15**), or 100% **P22-100** were yellow oils that were stable for extended periods of time; so far, over one year. A lower molecular weight analog **P21-25** was also prepared as a yellow oil. The rates of reaction were shown to be faster in more polar methanol (2 h) than in chloroform (12 h, Supporting Information).

**Figure 2:**
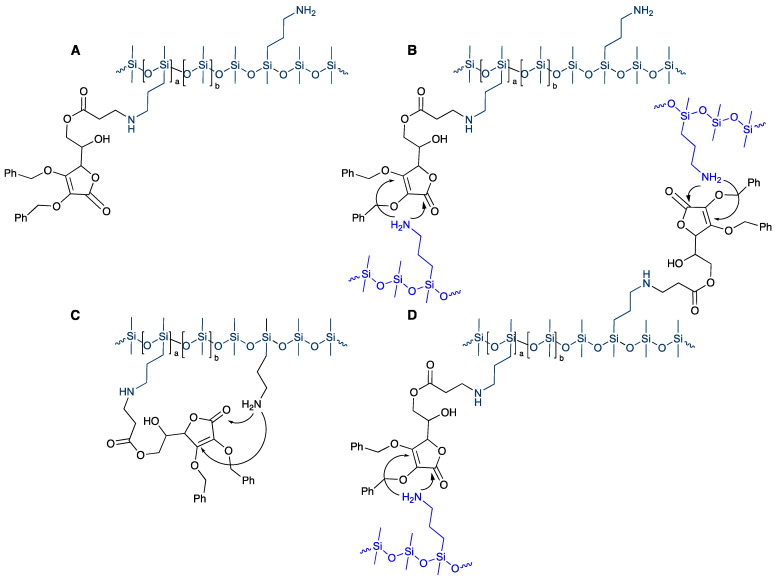
(**A**): monofunctional modifier; (**B**): chain extender; (**C**): loop reagent; or, at higher concentrations, (**D**): crosslinker.

**Figure 3:**
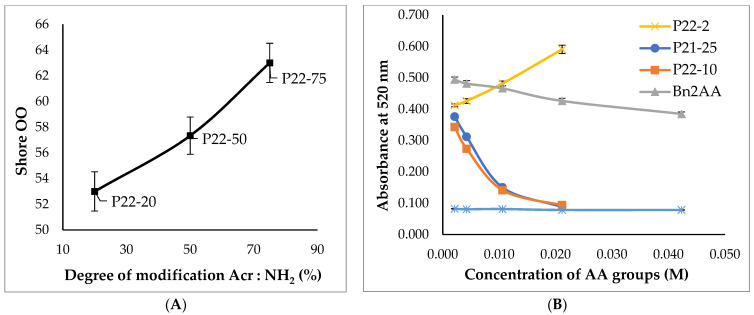
(**A**): Shore hardness data of benzylated ascorbic acid crosslinked aminoalkylsilicones for samples **P22-20**, **P22-50**, and **P22-75** (**B**): DPPH assay results showing antioxidant activity of ascorbic acid and benzylated ascorbic acid control compared with different debenzylated ascorbic acids.

If the higher molar mass pendent polymers based on **P22** were modified with higher quantities of **1** they ceased to be oils and were instead isolated as elastomers (**P22-20** ➞ **P22-75**). There was a direct correlation between the quantity of AA ‘crosslinker’ **1** added and the Shore hardness of the resulting elastomer, consistent with the formation of typical silicone elastomers [23,24]. The model study with butylamine suggests the origin of the observed crosslinking. When the stoichiometric excess of amines to acylates exceeds 1:1, the initial aza-Michael (similar to Figure 1C) is accompanied by a secondary reaction (similar to Figure 1D) where compound **1** bridges polymer chains leading to crosslinks analogous to **2**, **3**. One is obliged to explain, however, why there is an onset of elastomer formation only at 20% **1**. The silicone polymer has about 1 aminopropyl-containing monomer for each 5 D unit (Me_2_SiO). At low concentrations of **1**, the secondary reaction process will lead to both chain extension and intramolecular processes giving cycles (Figure 2B,C). In addition, not all the added **1** will undergo both processes. Thus, at lower concentrations of **1,** the aza-Michael reaction will lead to higher molecular weight silicone oils of viscosities that increase with the available fraction of **1**. At higher concentrations, sufficient crosslink arises that elastomers form, with crosslink density and durometer increasing in line with the relative quantity of **1** added. (Figure 3A).

### 3.3. Antioxidant Activity

The antioxidant activity of vitamin C is associated with the relative ease with which the ene-diol can undergo oxidation [25]. The ene-diols in products **T334**, **P21-25,** and **P22-x** were protected and, therefore, were not expected to have antioxidant activity. DPPH (2,2-diphenyl-1-picrylhydrazyl), a stable radical species, is a particularly convenient reagent for colorimetrically observing qualitatively, or determining quantitatively, antioxidant activity [26]. Neither **T334** nor any of the **P22-x** products exhibited significant antioxidant activity, as shown qualitatively when tested with 0.2 mM DPPH; over a period of 2 h the solution only very slowly turned yellow for oil samples, and 6–12 h for elastomeric samples, whereas ascorbic acid control solutions exhibited high antioxidant activity, immediately turning yellow. In quantitative DPPH assays, the benzylated ascorbic acid control also showed nearly no antioxidant activity (Figure 3B). It was inferred that, in order to reveal antioxidant activity, deprotection of the benzyl ethers to regenerate the ene-diol would first be necessary. 

**Figure 4:**
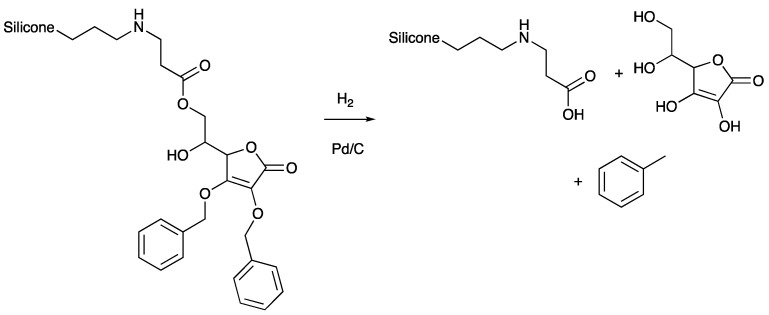
Reductive cleavage of benzyl ethers and the linking ester.

Benzyl ethers are conveniently cleaved by hydrogenation of Pd/C to yield the free alcohol and toluene. In our hands, the reduction process with both oils and elastomers led to the release of ascorbic acid (Figure 4) or its derivatives from the silicone. The reaction could be capricious; in one case, free ascorbic acid was isolated in an aqueous extract. More commonly, upon reduction of oils such as **P22-2**, **P22-10, P22-100,** or **P21-25** in IPA, the products took on a darker color and, in the case, of **P22-10** and **P21-25**, yielded black elastomers. That is, deprotection led to further crosslinking/chain extension. However, it also led to the liberation of antioxidant activity, as shown by DPPH assays (Figure 3B, Supporting Information). This suggests free enediols present in the product either as liberated ascorbic acid, or as tethered, crosslinking AA moieties.

## 4. Discussion

For the reasons articulated above, there remains much interest in the use of/release of vitamin C because of its powerful biological activities, including as an antioxidant. We do not consider materials in which vitamin C that is simply mixed into a matrix, and focus on materials in which the ascorbic acid is chemically grafted. There are surprisingly few examples of Vitamin C being used in a prodrug format. These include reports of the formation of esters of the ene-diol or at the C1 position to give materials that exhibit biological activity of various types after exposure to a biological environment. Proof of release of the ascorbic acid via ester hydrolysis is typically inferred. A vitamin C–ibuprofen ester was shown, for example, to cross the blood–brain barrier where a response to the ibuprofen was shown [27]; in this case, vitamin C was the carrier. Other examples describe the use of glycosides [28], or a combination of glycosides + aliphatic esters to link to vitamin C. In these cases, the biological release of vitamin C was reported after exposure to the spleen homogenates [29] or esterases [30]; in neither case was proof of the release of free vitamin C shown. In these examples the ester linkages operate, in part, to stabilize the vitamin C from degradation. 

Although vitamin C has been shown to be involved in various forms of crosslinking of polymers, particularly biological polymers, its role is generally to mediate the chemistry of the polymers themselves, including the crosslinking of proteins, for example, by inducing the Maillard reaction [31]. An important exception is the work of Leivo et al. who showed that the direct reaction of amine-modified silicone elastomer surfaces and with collagen permitted with ascorbic acid to link the two materials together via imines; the ascorbic acid moiety was then subject to autoxidative decomposition [19]. 

In the reactions described here, it is clear that—even when protected as benzyl ethers, **1** can undergo at least 2 sequential reactions under mild conditions (Figure 1C,D and Figure 2) leading first to chain extension and then crosslinking to give robust silicone elastomers that do not have antioxidant activity—the enediol is protected. This form of vitamin C is thus a convenient, natural crosslinking agent.

Upon liberation of the enediol by reductive deprotection of the benzyl ethers further crosslinking ensued. The accompanying darkening in color is consistent with a Maillard reaction. Elaborating the subtleties of these processes is a current occupation. Regardless, the products are also efficient antioxidants whether free ascorbic acid is liberated, or the crosslinker retains the enediol. 

Simple silicone fluids undergo rather efficient environmental depolymerization [32,33]. While speculation only, it is expected that silicone fluids modified by **1**, and elastomers formed following reductive deprotection, will be subject to ester hydrolysis to regenerate silicon oils (Figure 4) that will also undergo facile depolymerization. The new crosslinks formed by ascorbic acid self-reaction should be analogous to the normal outcomes of ascorbic acid self-condensation and should also be readily degraded. The conditions for reductive cleavage of benzyl ethers are mild, but require the transition metal catalyst for efficient cleavage. This is an aspect that is clearly disadvantageous. However, it may be possible to elicit reductive cleavage without the need for platinum; it is noted that some benzyl ethers are susceptible to both oxidative and reductive cleavage under biological conditions [34].

The Green Chemistry rules call for materials that make better use of natural feedstocks [35]. In the present case, while **1** does dilute the synthetic silicone, it is to a small extent only (and we note that not all aspects of the synthesis are consistent with Green Chemistry, e.g., the (de)protection sequences). However, the use of vitamin C provides both a useful mechanism for crosslinking and delivery of new functionality—natural antioxidant activities—during cleavage. We hope to demonstrate this utility will be accompanied by the more facile decomposition of the silicone component at end of life. 

## 5. Conclusions

Benzyl-protected ascorbic acid-modified silicones were successfully synthesized using an aza-Michael addition; the ascorbic acid ranged from 2% to 100% on both telechelic and aminoalkylsilicones. The ascorbic acid acts as a crosslinker for pendent silicones to give robust silicone elastomers without significant antioxidant behavior. Reductive debenzylation was expected to liberate antioxidant activity but, surprisingly, also lead to cleavage of the crosslink to give silicone oils and vitamin C. Thus, aspects of this work: natural materials, function, and programmed degradation fall within the rules of Green Chemistry. 

## Data Availability

Spectroscopic data may be found in the Supporting Information.

## Supplementary Materials

The following supporting information can be downloaded at: https://www.mdpi.com/article/10.3390/polym14225040/s1, Table S1: quantities for polymer synthesis, and Tables S2 and S3 for DPPH analyses; Figures S1–S10 ^1^H, ^13^C NMR spectra and 1 mass spectrum of starting materials and silicone products; and Figure S11 photo of DPPH assay.
