# Supplementary material for: Ascorbic Acid-Modified Silicones: Crosslinking and Antioxidant Delivery
Source: Polymers (Basel). 2022 Nov 21;14(22):5040. doi: 10.3390/polym14225040 (PMC9693009; doi:10.3390/polym14225040)
Supplement: Supplementary file 1 [file polymers-14-05040-s001.zip › polymers-2006309-supplementary.pdf]

Ascorbic Acid-Modified Silicones: Crosslinking and Antioxidant Delivery

Guanhua Lu [1], Akop Yepremyen [1], Khaled Tamim [1], Yang Chen [1] and Michael A. Brook [1,*]

Supporting Information

Table S1. Experimental details of the synthesis of benzylated, acryl ascorbic acid -modified silicones.

Sample Name	% ACR to NH_2	mass of P-22 (g)	Volume of 0.02 g/mL Bn_2Asc stock solution
**P22-2**	2.00%	2.46	2.5
**P22-5**	5%	0.99	2.5
**P22-10**	10%	0.49	2.5
**P22-15**	15%	0.33	2.5
**P22-20**	20%	0.25	2.5
**P22-50**	50%	0.10	2.5
**P22-75**	75%	0.07	2.5
**P22-100**	100%	0.05	2.5
**P21-25**	25%	1.04	2.5

Table S2 DPPH Assay sample preparation weight

**P21-25**		**P22-2**		**P22-10**	
AA group concentration (M)	Elastomer Weight (g)	AA group concentration (M)	Elastomer Weight (g)	AA group concentration (M)	Elastomer Weight (g)
0.0212	0.0500	0.0212	0.0500	0.0212	0.0500
0.0106	0.0250	0.0106	0.0250	0.0106	0.0250
0.0042	0.0100	0.0042	0.0100	0.0042	0.0100
0.0021	0.0050	0.0021	0.0050	0.0021	0.0050

Table S3 DPPH Assay result summary

	Absorbance at 520 nm (n=3)									
	**P22-10**		**P22-2**		**P21-25**		**Bn2AA**		AA	
AA Conc. (M)	Average	Std Error	Average	Std Error	Average	Std Error	Average	Std Error	Average	Std Error
0.002	0.343	0.006	0.412	0.005	0.376	0.005	0.494	0.008	0.082	0.001
0.004	0.274	0.004	0.426	0.008	0.312	0.003	0.481	0.010	0.081	0.001
0.011	0.141	0.002	0.482	0.008	0.150	0.003	0.466	0.009	0.081	0.002
0.021	0.094	0.002	0.591	0.013	0.088	0.001	0.426	0.009	0.078	0.000
0.042							0.385	0.006	0.078	0.001

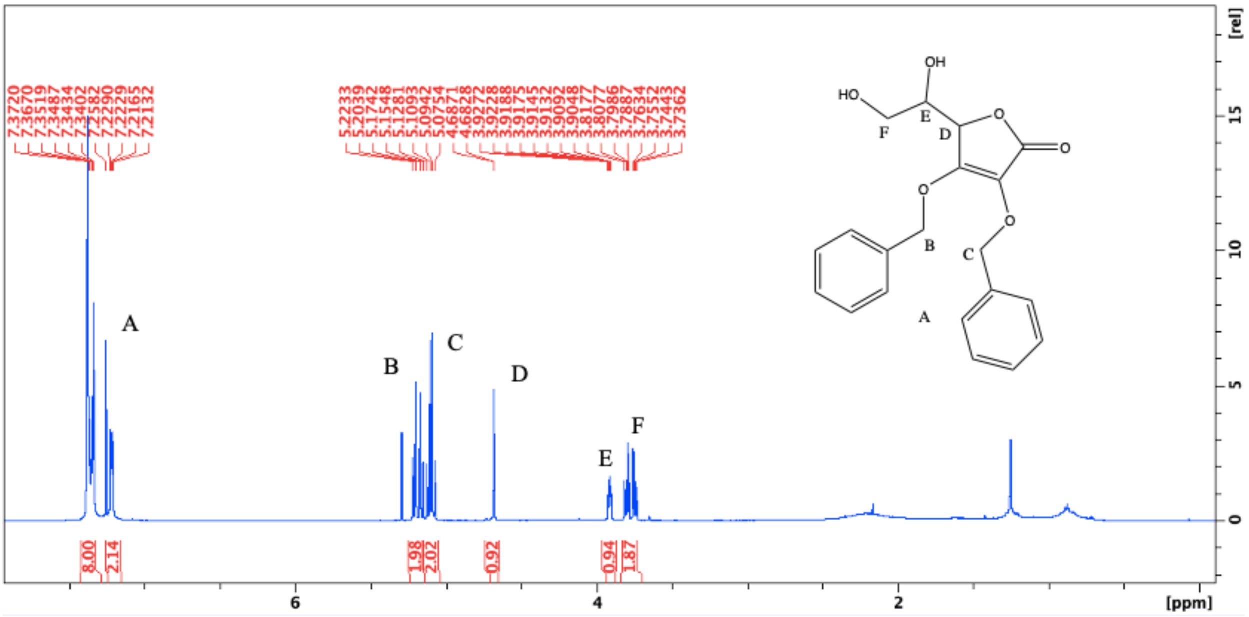

Figure S1. ^{1}H NMR spectrum of benzylated ascorbic acid **Bn2AA**.

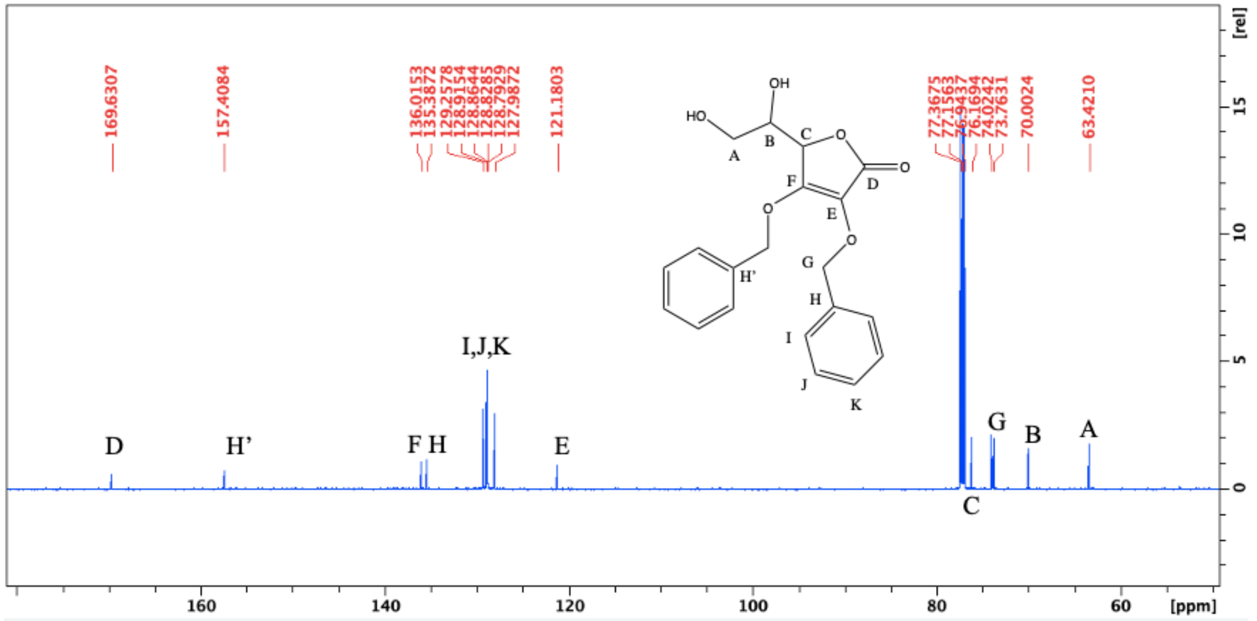

Figure S2. ^{13}C NMR spectrum of benzylated ascorbic acid **Bn2AA**.

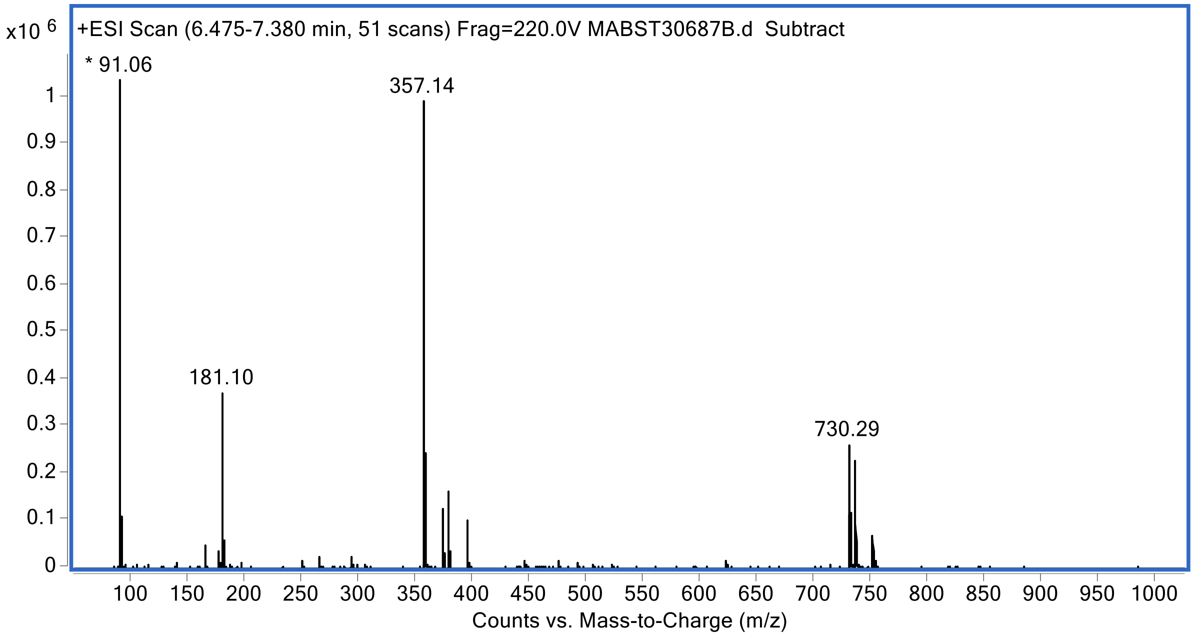

Figure S3. Mass spectrum (ESI, Ve+ mode) of benzylated ascorbic acid Bn2AA. Shown [M+H]+, [M+NH4]+, [M+Na]+ and [M+K]+ at m/z 357, 374, 379 and 395 respectively.

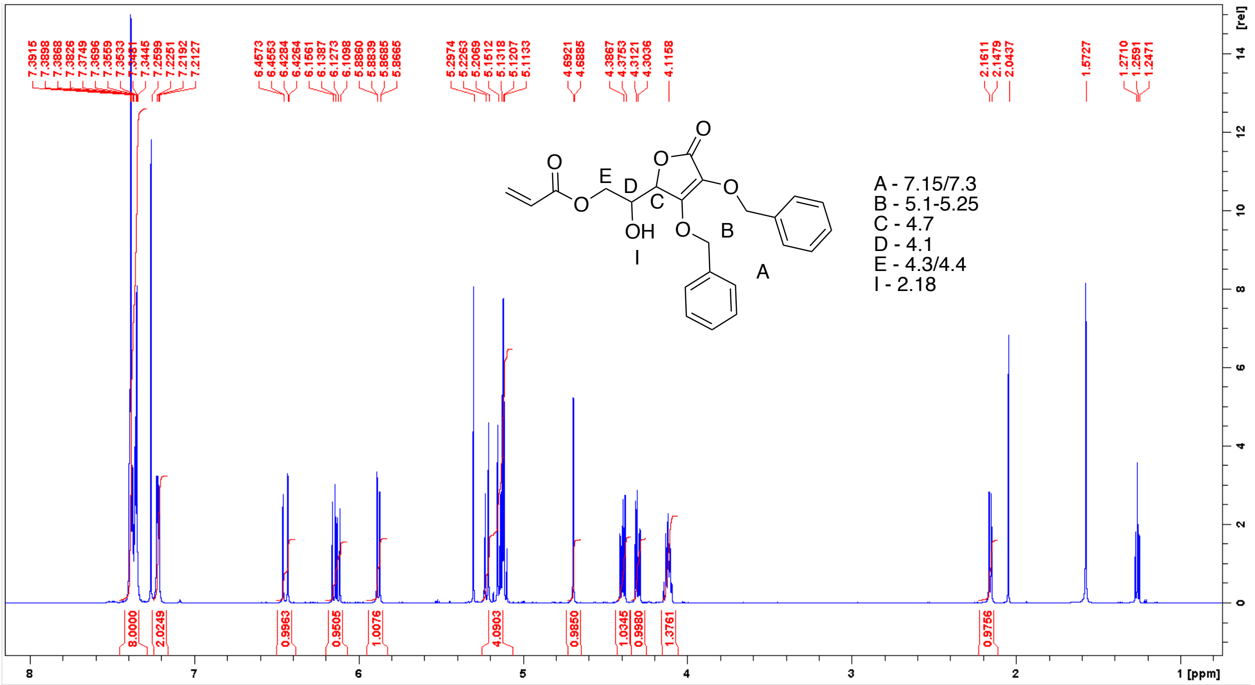

Figure S4. ^{1}H NMR of benzylated acryl ascorbic acid **1.**

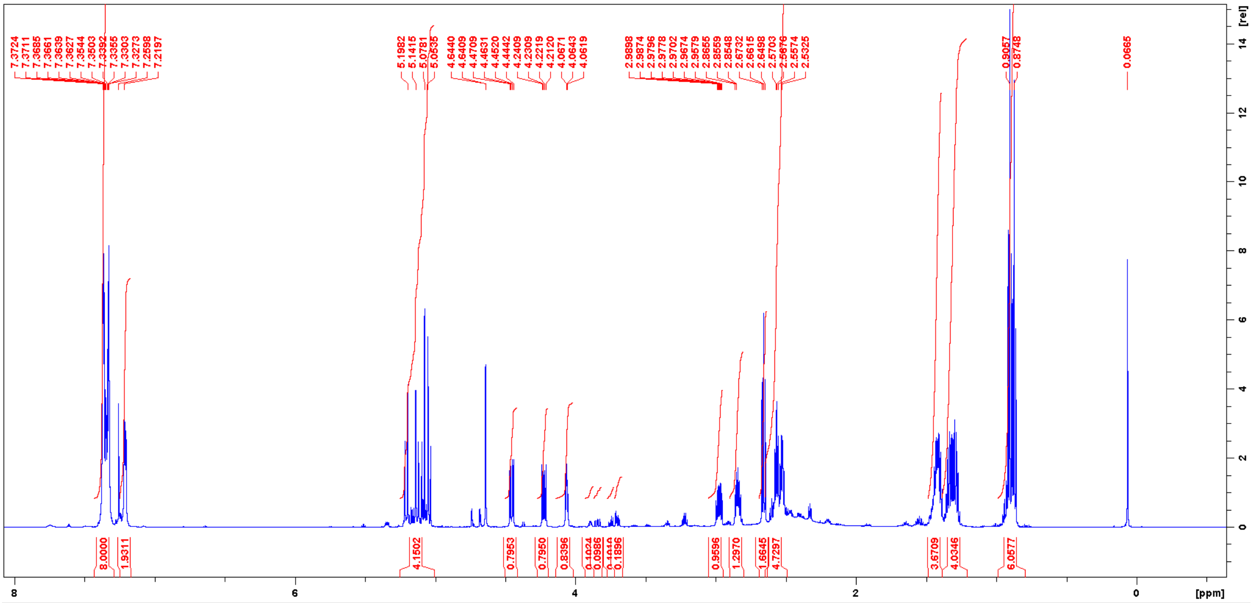

Figure S5. Final product of benzylated acryl ascorbic acid with over excess butyl amine, shown two eq butyl amine was reacted with benzylated acryl ascorbic acid **N2AA**.

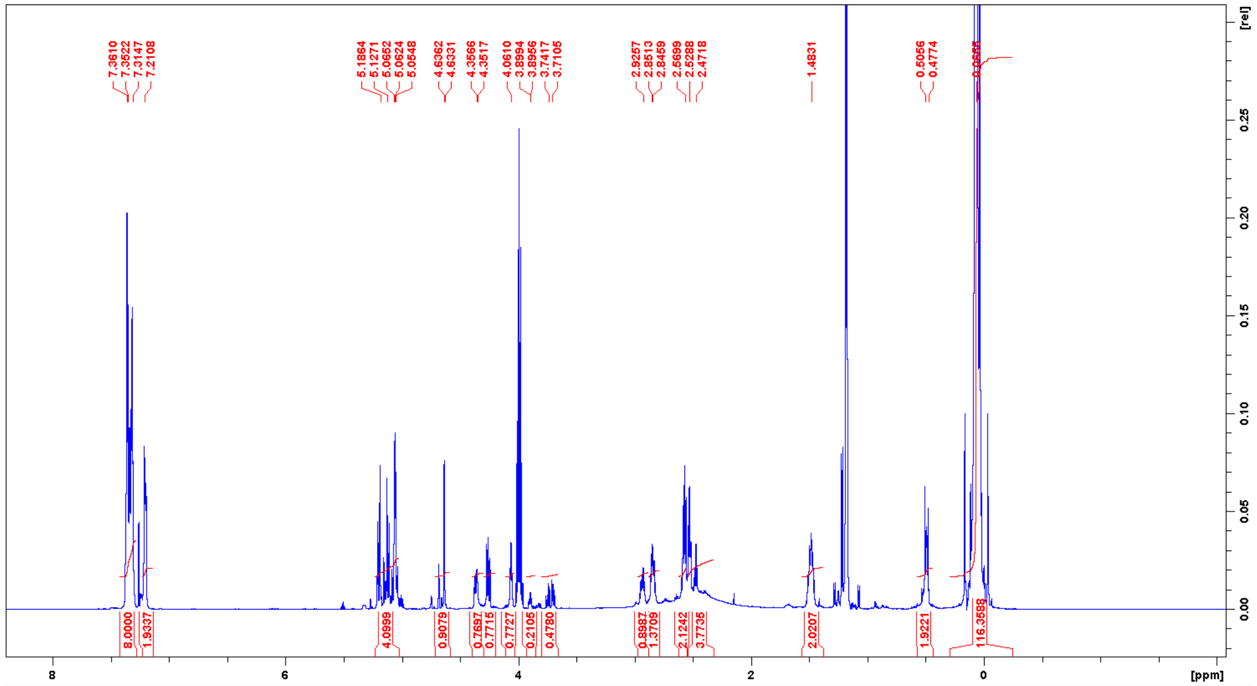

Figure S6. Benzylated acryl ascorbic acid **1**-modified **T334** reaction in IPA after overnight.

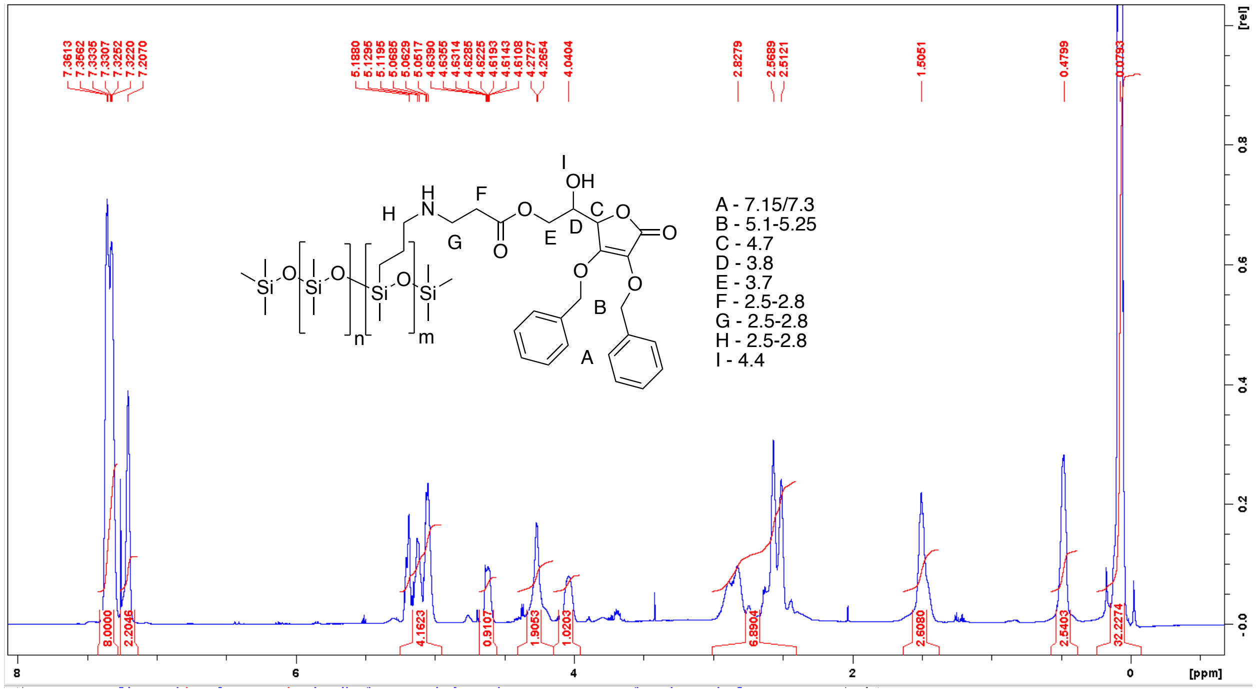

Figure S7 ^{1}H NMR of benzylated acryl ascorbic acid modified **P22-100** reaction in $CDCl_3$ after 24h.

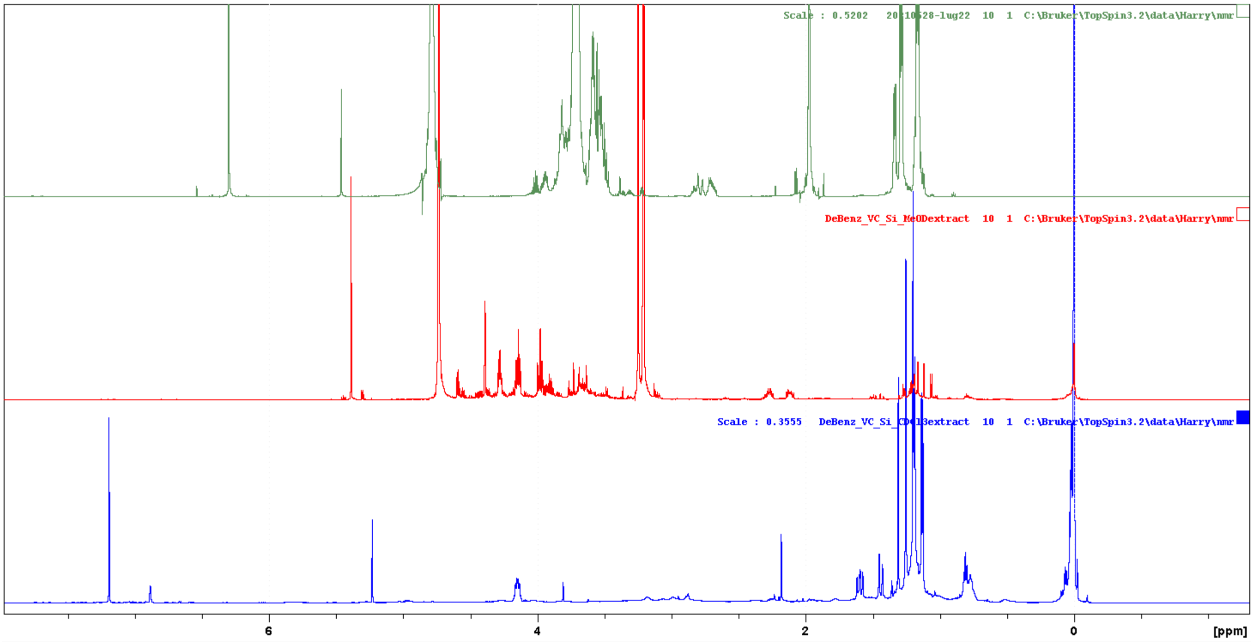

Figure S8. Hydrogenation product of **P22-100** from bottom to top to be $CDCl_3$ extract, MeOD-d_4 extract and D_2O extract.

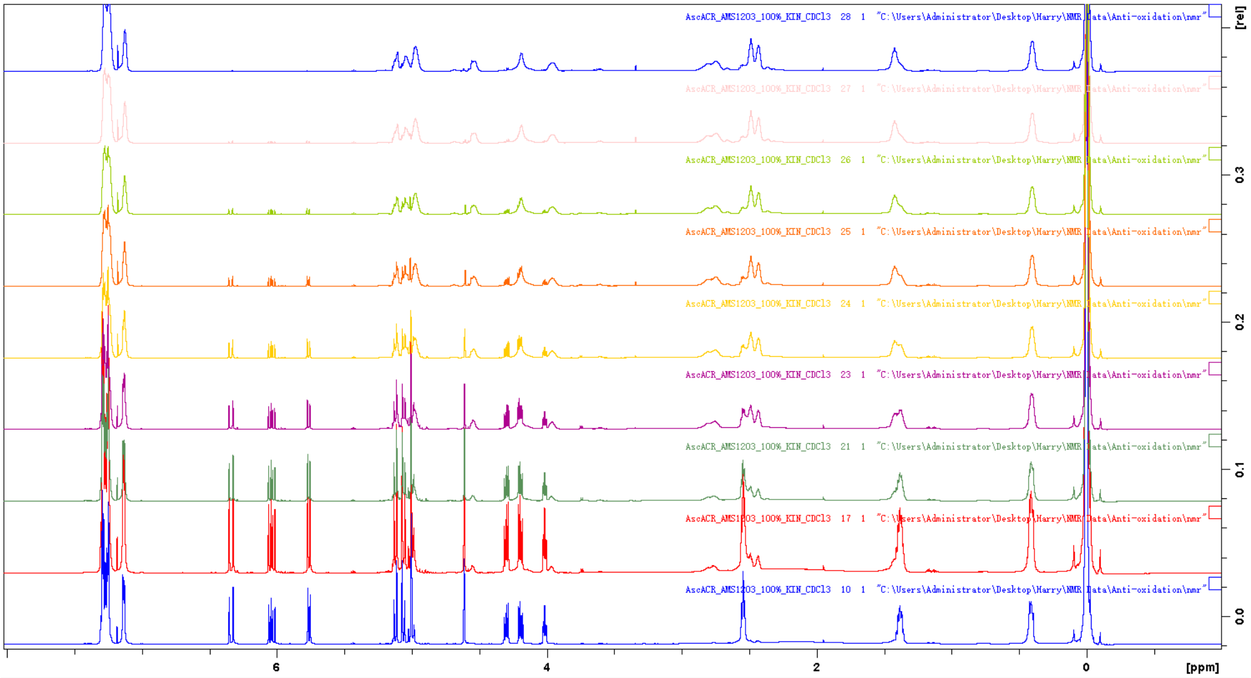

Figure S9. Kinetic study of benzylated acryl ascorbic acid **1** reacting with **P22** in $CDCl_3$. Reaction time 0, 15 min, 30 min, 1 h, 2 h, 4 h, 8 h, 12h, 24 h from bottom to the top.

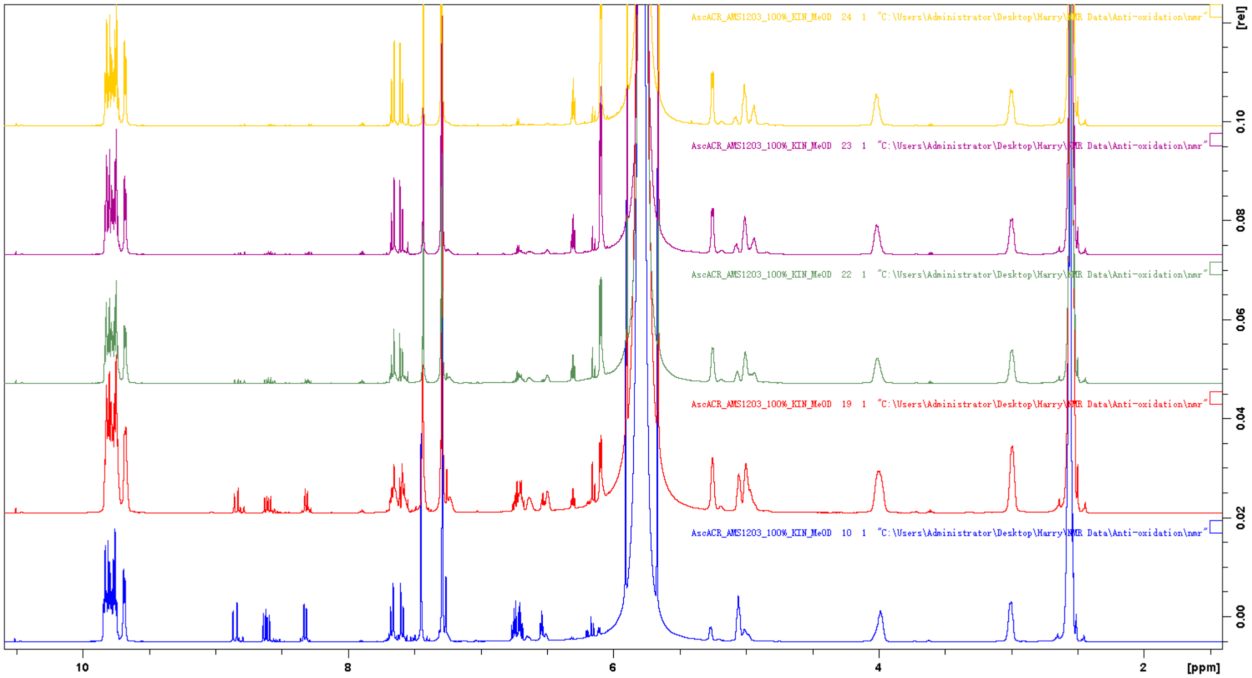

Figure S10. Kinetic study of benzylated acryl ascorbic acid **1** reacting with **P22** in MeOD-d_1. Reaction time 0, 15min, 30min, 1h, 2h from bottom to the top.

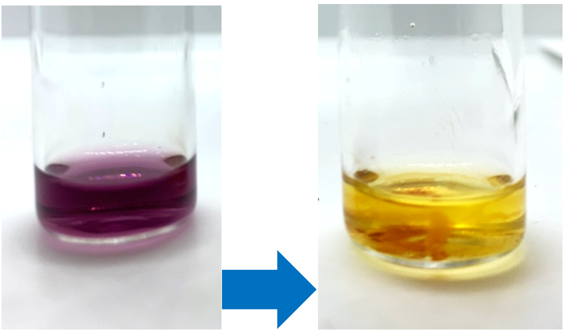

Figure S11. Antioxidant testing: hydrogenated product mixture of **P22-100** before and immediately after addition of DPPH.
